# The association between passive social network usage and depression/negative emotions with envy as a mediator

**DOI:** 10.1038/s41598-023-37185-y

**Published:** 2023-06-21

**Authors:** Wen Cheng, Duc Nhan Nguyen, Pham Ngoc Thien Nguyen

**Affiliations:** 1 Center for Teacher Education, Institute of Education, International Graduate Program of Education and Human Development, National Sun Yat-Sen University, Kaohsiung, Taiwan, ROC; 2 International Graduate Program of Education and Human Development, National Sun Yat-Sen University, Kaohsiung, Taiwan, ROC; 3 Ho Chi Minh City University of Education, Ho Chi Minh City, Vietnam; 4 An Giang University, Vietnam National University Ho Chi Minh City, Ho Chi Minh City, Vietnam

**Keywords:** Psychology, Risk factors

## Abstract

This study aimed to investigate the relationships between passive social network usage (PSNU) and depression/negative emotions over time with the mediating role of envy among Vietnamese adolescents. First, it revealed that PSNU had a simultaneous effect on depression/negative emotions as well as at different time points, indicating that social network site behaviors can predict psychological states over time (explained by the social comparison theory). Second, the autoregressive effect also confirmed a potential reciprocal relationship between PSNU and depression, whereas PSNU appeared to have an impact on negative emotions but not the other way around. Specifically, depression at Time 1 was positively associated with PSNU at Time 2, whereas negative emotions did not exhibit a similar pattern (explained by the cognitive dissonance theory). The different associations were interpreted as depression having cognitive elements, while negative emotions were thought to be purely emotional states. The results demonstrated that behavior may potentially have a long-lasting effect on mental health (both negative emotions and depression), while it was depression, rather than negative emotions, that had a long-term effect on behaviors. Third, envy played a mediating role that connected the changes of PSNU and depression/negative emotions. The implications and limitations of these findings are discussed.

## Introduction

Social networks or online social networks, which have developed thanks to the growth of the internet, are groups of people linked by relationships derived from data about their activities, shared communication, and often known as a kind of virtual societies^1^. Over the last decade, social networking sites (SNSs) have become a huge internet phenomenon^2^, and an integral component of human interactions^3^, especially among the young generation^4,5^. Some research has revealed that people utilize social media networks to alleviate stress, anxiety, and depression, improve their mood, obtain comfort and joy, and prevent loneliness^6,7^. On the other hand, there have been cross-sectional studies focusing on the negative effects of social media use on people’s wellbeing and mental health^3,8^. The concern for therapists is to be proactive in filling gaps in mental illness prevention^9^, especially long-term effects on mental health. However, to the best of our knowledge, scant research has adopted the longitudinal research approach to examine the mediating effect of envy on the relationship of passive social network usage (PSNU) with both depression and negative emotions. Following the direction, our study, adopting a longitudinal approach, aims to clarify how PSNU is connected to depression/negative emotions via envy at every time point and how depression/negative feelings may have a long-term impact on SNS usage, particularly PSNU.

Most studies on social network usage and psychological outcomes have been conducted with undergraduates as participants, i.e.,^2,6,10,11^. However, social network usage is also prevalent among high school students^12^. Although there have been some research revealed social networking site usage was linked with negative outcomes, e.g., addiction tendencies, fatigue, anxiety, low learning performance, skipped classes, and poor relationships with peers^13–15^, research focusing on passive social media use and psychological outcomes in adolescents is still limited. More research on the benefits and drawbacks of PSNU, including its potential causal relationship with mental health issues, is required to provide a scientific-based evaluation of this popular behavior among high school students.

## Passive social network usage (PSNU) and negative emotions

### Passive social network usage (PSNU)

*Passive social network users* are people who use social media but rarely engage in communication and interaction with others^16^. Passive social network usage (PSNU) is considered a possible reason for some mental health problems in the young generation^17^, and it has been shown to correlate with symptoms of anxiety and depression^5,18^.

In addition, passive social network users are more likely to experience negative social comparison and negative affect^2^. Multiple studies have linked passive social media use with low subjective wellbeing^16,19^, reduced life satisfaction^3,20^, and increased negative emotions^21^. PSNU has been found to be related to other negative outcomes, such as envy, which affects the cross-sectional relationship between PSNU and depression^6^. Specifically, when envy was evoked, using Facebook for surveillance was found to be positively related to depression^6^. Therefore, it is critical to study how PSNU affects individuals’ mental health and, in the current study, how it relates to negative emotions and depression. By understanding such relationships might allow the development of interventions or preventative strategies for at-risk populations.

### Depression and negative emotions

As mentioned, negative emotions or depression are associated with PSNU. Negative emotions are typically characterized as distressing or unhappy feelings that exert a deleterious impact on individuals, leading to feelings of loneliness, depression, anxiety, and anger^22^. On the other hand, depression is defined as a pervasive sense of hopelessness, discouragement, or irritability, which can lead to a variety of emotional and physical problems, thereby impairing one's functionality both at work and home. Symptoms may include memory problems, chronic body aches, or concentration difficulties^23^. As posited by Beck^24^ and Aekwarangkoon and Thanathamathee^9^, depression can be triggered by a cognitive process marked by negative automatic thoughts, fostering pessimistic perceptions of oneself, the world, and the future.

Negative emotions and depression are prevalent mental health concerns among adolescents^25,26^. Specifically, negative emotions may escalate during late adolescence, with interpersonal relationship conflicts potentially becoming more frequent and impactful during this phase, thereby impairing adolescents' capacity for emotion regulation^26^. Although negative emotions may appear less detrimental than depression, the latter can potentially yield more severe repercussions, such as suicide^26–28^. Research delineates that negative emotions (e.g., fear, anxiety) and depression in adolescents, while distinct, are correlated^29^. Studies suggest that depression can coexist with negative emotional traits^10,26,30^. Given the nuanced relationship between depression and negative emotions, research focused on adolescents should encompass both to present a comprehensive understanding of these mental health issues' potential impact on behavior. In the current study, we approach negative emotions as emotional states (which may be caused by or result in cognitive processes), while we regard depression as states explicitly incorporating both emotional and cognitive dimensions.

Considering upward social comparisons^31^, passive social network users who frequently peruse others' SNS profiles may feel that others are more content or successful than they are, leading to feelings of envy towards their peers^6,18^. In other words, PSNU may incite envy, which may subsequently predict depression or negative emotions. In addition to its emotional aspect, envy also encompasses a cognitive component^32,33^ that may contribute to depression. The social comparison theory may shed light on the relationship between PSNU and negative emotions/depression, with envy acting as a mediator.

### Upward social comparisons

Festinger^31^ proposed the social comparison theory, which argues that people have an inbuilt desire to evaluate themselves in comparison to others. Social networking platforms are an ideal media for users to engage in social comparison, and both upward and downward comparisons are related to mental health^11,34^. Verduyn, et al.^34^ argued that most social networking sites encourage upward social comparisons, which may lead to envy. Furthermore, previous research has shown that PSNU users are more likely related to upward social comparisons, rather than downward social comparisons^2,21,35^. According to the social comparison theory, upward social comparison occurs when people compare themselves with others they perceive as superior^36^, which may induce more negative feelings^37^. Upward social comparisons on SNSs may be unavoidable. People engaged in PSNU will compare themselves with others, especially those who are similar to them^38^, which make the individuals feel envious of others with perceived better lives^2^. In addition, a longitudinal study demonstrate that PSNU can be indirectly associated with depressive symptoms via evoking envy^18^.

Envy is one of a collection of emotions including humiliation, jealousy, relative deprivation, and indignation, and is defined by negative affective reactions to others’ greater fortunes^39,40^. Because envy is the experience of a self-threatening feeling, it often promotes aggressive behaviors that involve hostile emotions^32^ or are devoid of positive feelings^33^.

In a sense, envy violates social harmony conventions which usually require people to support one another rather than compete with or react to another person’s success, and it has been revealed to link with negative mental health outcomes^7,16^. Feelings of envy can be triggered by overexposure to social information on SNSs and eventually predicted significant damage to users’ wellbeing and life satisfaction^20,32^.

Existing studies found significant relationships between social network usage and wellbeing^16,21^ and between PSNU and depression^18^ as well as negative emotions^17^. However, the previous research largely focused on cross-sectional studies^17,41^; thus, longitudinal evidence is needed to understand how the reciprocal relationships between PSNU and depression/negative emotions have changed over time. Particularly, longitudinal research can be adopted to understand the long-term effects and possible causality of the variables. Thereby, the causal inferences regarding the relationships of the constructs in the hypothesized model might be verified. This can help explain how individuals’ PSNU associates with their negative feelings or depression reciprocally.

Thus, the current study assessed the same participants’ self-reported PSNU, negative emotions, depression, and envy, at two different time points (Time 1 and Time 2, two months apart). The mediating role of envy in the associations between PSNU and negative emotion/depression would be examined in both different times. The hypotheses are the following:

#### Hypothesis 1

*Hypotheses 1.1 & 1.2.* Envy may play a mediating role, in the relationship between PSNU and depression at Time 1 (*Hypothesis 1.1*) and at Time 2 (*Hypothesis 1.2*). More precisely, PSNU may trigger envy, which, in turn, may be linked to an increase in depression.

#### Hypothesis 2

*Hypotheses 2.1 & 2.2.* Envy may play a mediating role, in the relationship between PSNU and negative emotions at Time 1 (*Hypothesis 2.1*) and at Time 2 (*Hypothesis 2.2*). More precisely, PSNU may trigger envy, which, in turn, may be linked to an increase in negative emotions.

#### Hypothesis 3

*Hypothesis 3.1.* PSNU at Time 1 may be linked to depression at Time 2.

*Hypothesis 3.2.* PSNU at Time 1 may be linked to negative emotions at Time 2.

The relationship between PSNU and depression/negative emotions is expected to be influenced by upward social comparison^2,18^. The goal of this research is to examine the reciprocal relationships; therefore, the relationship between depression/negative emotions and PSNU needs to be examined next. The second framework for consideration is cognitive dissonance theory.

### Negative emotions/depression in PSNU: the cognitive dissonance theory^42^

Emotional states have a significant impact on how people perceive and engage in their environments. *Cognitive dissonance theory* proposes that people tend to keep their attitudes and behaviors in sync and prevent dissonance which causes discomfort^42,43^.

According to the cognitive dissonance theory^42^, people seek for cognitive equilibrium or solutions to decrease their tensions^44^. Rooted in the cognitive dissonance theory^42^, selective exposure suggests that people would continue to look for information consistent with their preexisting cognitions or attitudes in order to reduce potential future cognitive dissonance^44^. It is plausible to assume that when individuals experience in a bad mood, they are more likely to passively utilize SNSs which may also produce the negative emotions, in order to be consistent with their preexisting psychological states.

Building on the cognitive dissonance theory^42^, individuals strive for cognitive equilibrium and seek ways to reduce internal tension. The concept of selective exposure posits that people tend to seek information that aligns with their pre-existing cognitions or attitudes to minimize potential future cognitive dissonance^44^. Individuals experiencing depression or negative emotions might engage in activities as a means to alleviate cognitive dissonance arising from conflicting beliefs and behaviors, and to maintain congruence with their pre-existing psychological states^42^. Therefore, it is hypothesized that individuals experiencing depression or negative emotions at Time 1 may harbor negative self-perceptions or outlooks on life. Passive social network usage, characterized by browsing social media feeds without actively participating in conversations or sharing content^5^, could present opportunities for social comparison that aggravate these negative beliefs and intensify cognitive dissonance. Consequently, individuals may escalate their passive social network usage at Time 2 in an attempt to mitigate cognitive dissonance and maintain alignment with their pre-existing mood states, such as depression and negative emotions. Thus, the increase in passive social network usage at Time 2 may be considered as a coping mechanism driven by the cognitive dissonance experienced by individuals with depression or negative emotions. Thus, we hypothesize the following:

#### Hypothesis 4

*Hypothesis 4.1.* Depression at Time 1 may be positively linked to PSNU at Time 2.

*Hypothesis 4.2.* Negative emotions at Time 1 may be positively linked to PSNU at Time 2.

In sum, based on the upward social comparison^31^, we proposed that envy may play a role in mediating the relationships between PSNU and depression/negative emotions. Moreover, the bidirectional relationships between PSNU and depression/negative emotions were also examined based on both the social comparison theory^31^ and the cognitive dissonance theory^42^, in order to determine the possible route of causality.

## Methods

### Participants and procedure

The data in the current study were collected at two different time points on high school students in southern Vietnam. The first wave of data collection took place over approximately two weeks starting in early January 2021 (Time 1), including 576 high school respondents. The second wave of data collection was from the late February to early March 2022 (Time 2), with the same sample participants. However, the attrition rate was 7.6% (44 students completing only at Time 1), resulting in the total number of two-time completions as 532 participants. At Time 1, the number of male and female students was 308 and 268 (53.5% and 46.5%), respectively; and at Time 2, the number of male and female students was 291 and 240 (54.7% and 45.1%). The percentages of the participants with two-time completions of grades 10, 11, 12, which were 35.0%, 53.6%, and 11.0%, respectively. Detailed backgrounds information of participants is provided in Table 1.

**Table 1:** Descriptive statistics of the study variables.

Variables	Level	*M*	*SD*	Range	*N*	Percentage (%)
Time 1 (*N* = 576)						
Sex	Male				308	53.5
	Female				268	46.5
Grade	10				197	34.2
	11				315	54.7
	12				64	11.1
PSNU		2.60	0.62	1.00–4.80	576	
Envy		2.94	0.65	1.00–4.88	576	
Depression		2.02	0.48	1.10–3.65	574	
Negative emotions		2.72	0.67	1.00–5.00	576	
Time 2 (*N* = 532)						
Sex	Male				291	54.7
	Female				240	45.1
	Missing				1	0.2
Grade	10				186	35.0
	11				285	53.6
	12				59	11.0
	Missing				2	0.4
PSNU		3.02	0.78	1.00–5.00	532	
Envy		2.94	0.64	1.00–5.00	532	
Depression		2.05	0.47	1.10–3.60	527	
Negative emotions		2.71	0.64	1.00–5.00	529	

*PSNU* passive social network usage.

The questionnaire mentioned below was revised and translated from English into Vietnamese. First, the English questionnaire was converted into Vietnamese. Subsequently, an independent researcher translated the Vietnamese version back into English and compared it with the original English version to verify the accuracy of the items' meanings. Finally, the other three language lecturers gave advice to enhance the translation accuracy and ensure its context-based meaning.

The participants were from four high schools in urban areas in southern Vietnam where social media is popular among adolescents. The distribution of the questionnaire was approved by the high school administrator. The questionnaire was sent to the instructor of each class. Participants were recruited to fill out the survey voluntarily. After reading the informed consent and instruction regarding the study, the participants took approximately 20 min to complete the survey. Paper-and-pencil survey was used to collect data for Time 1; either paper-and-pencil or online format (71.8% and 28.2%, respectively) was used to collect data for Time 2. The American Psychological Association's research ethics standards were implemented for this study. The informed consent forms from participants and parents/participants’ legal guardians were included before the survey started.

### Measures

#### Passive social network usage (PSNU)

To measure the behavior of using social network of participants, PSNU was assessed by five items adapted from^21^, excluding one item with low consistency (i.e., “How many times (on average) do you have your social networking sites content updated every day?”). One of the sample items used was “How often (on average) do you look through your friends’ social networking sites homepage*?* Participants were asked to evaluate their opinions on a 5-point Likert scale, with 1 = *never* and 5 = *almost every time you open social networking sites.* The internal consistency reliabilities of Time 1 and Time 2, which were *α* = 0.63 and *α* = 0.75, respectively, were satisfactory^45^. The higher mean scores indicated the higher levels of PSNU.

#### Envy

Facebook Envy Scale was utilized to assess one’s envious feelings^6^. The scale included eight items which measure how much the participants compared themselves with others on a 5-point Likert scale ranging from 1 = *strongly disagree* to 5 = *strongly agree.* A sample question was “*Many of my friends have a better life than me.*”. The internal consistency reliabilities of the current study at Time 1 and Time 2 were *α* = 0.71 and *α* = 0.73, respectively. Higher mean scores indicated higher levels of envy.

#### Depression

The Center for Epidemiologic Studies Depression Scale is a 20-item instrument used to measure an individual’s level of depression^46^. The scale consists of emotion (e.g., “I was happy”) and cognition items (e.g., “I had trouble keeping my mind on what I was doing”). Each of the 20 items is rated on a 5-point Likert scale ranging from 1 = *rarely or none of the time* (*less than 1 day*) to 5 = *Most or all of the time* (*3–7 days*)*.* The positively worded statements were coded reversely before the mean calculations were conducted. The internal consistency reliabilities of the current study at Time 1 and Time 2 were *α* = 0.83 and *α* = 0.85, respectively. Higher mean scores revealed higher levels of depression.

#### Negative emotions

Negative emotions were measured by using The Positive and Negative Affect Schedule (PANAS)^47^. Ten items assessing negative emotions (e.g., afraid, upset) were rated on a 5-point Likert scale ranging from 1 = *very slightly or not at all* to 5 = *extremely.* The internal consistency reliabilities of the current study at Time 1 and Time 2 were *α* = 0.83 and *α* = 0.85, respectively. Higher mean scores revealed higher levels of negative emotions.

### Data analysis

PROCESS Macro for SPSS version 3.4 (Model 4)^48^ was used to test the mediating effect of envy in the relationship between PSNU and depression as well as negative emotions, at both time points (Time 1 and Time 2). To test the significance of mediation effects, the bias-corrected percentile boostrap method based on 5000 bootstrapping samples was used. Moreover, the cross-lagged model in path analysis formats was conducted via AMOS in order to examine the reciprocal relationship between PSNU and depression/negative emotions. As a diverse way to Pearson’s correlations.

## Results

### Descriptive statistics for high school participants

The descriptive statistics displays in Table 1.

The correlations of variables are presented in Table 2. It showed that all the variables at Time and at Time 2 were positively correlated with each other.

**Table 2:** Pearson’s correlations between study continuous variables.

Variables	1	2	3	4	5	6	7
1. PSNU at T1	–						
2. Envy at T1	0.21***	–					
3. Depression at T1	0.13**	0.46***	–				
4. Negative emotions at T1	0.24***	0.33***	0.57***	–			
5. PSNU at T2	0.43***	0.20***	0.15***	0.11*	–		
6. Envy at T2	0.26***	0.47***	0.39***	0.28***	0.22***	–	
7. Depression at T2	0.15***	0.28***	0.44***	0.27***	0.18***	0.42***	–
8. Negative emotions at T2	0.19***	0.14***	0.30***	0.42***	0.28***	0.30***	0.53***

*PSNU* passive social network usage, *T1* Time 1, *T2* Time 2. As a diverse way to consolidate reliability of the used scales, test–retest reliability was applied (i.e., PSNU, depression, negative emotion, and envy) with the same instrument at Time 1 and Time 2, indicating that the associations between the two variables between Time 1 and Time 2 were quite stable and consistent (see Table 2).

*p < 0.050, **p < 0.010, ***p < 0.001.

### Testing the mediating effect of envy and the cross-lagged models of PSNU and depression

#### Testing the mediating effect of envy

PSNU positively predicted envy (Time 1: *β* = 0.21, *p* < 0.001; Time 2: *β* = 0.22, *p* < 0.001), envy positively predicted depression (Time 1: *β* = 0.45, *p* < 0.001; Time 2: *β* = 0.40, *p* < 0.001), and the total effect of PSNU on depression without envy was significant (Time 1: *β* = 0.10, *p* = 0.001; Time 2: *β* = 0.18, *p* < 0.001); but it turned to be nonsignificant when envy was controlled, Time 1: *β* = 0.04, *p* = 0.269 and it was still significant when envy was added, Time 2: *β* = 0.09, *p* = 0.026.

The indirect effect of envy was estimated, Time 1: 0.07, 95% CI = [0.04, 0.10], and Time 2: 0.06, 95% CI = [0.04, 0.10], indicating that envy was a full mediator at Time 1 but it was only a partial mediator in the association between PSNU and depression at Time 2. The effect size of envy was estimated at Time 1 (κ^2^ = 0.096, 95% CI = [0.06, 0.14]) and at Time 2 (κ^2^ = 0.091, 95% CI = [0.05, 0.13]), indicating medium effect sizes^49,50^. Therefore, *Hypotheses 1.1 and 1.2 were supported*.

#### Testing the cross-lagged model of PSNU and depression

**Figure 1:**
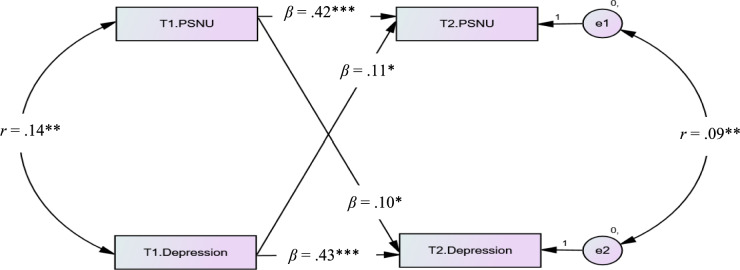
The mediating effect of envy and cross-lagged model of PSNU and depression.* PSNU* passive social network usage. The significant paths were showed by the solid lines. *T1* Time 1, *T2* Time 2.*p < 0.050., **p < 0.010, ***p < 0.001.

PSNU at Time 2 was predicted by depression at Time 1 (*β* = 0.11, *p* = 0.013) after PSNU at Time 1 was controlled (*β* = 0.42, *p* < 0.001). On the other hand, depression at Time 2 was predicted by PSNU at Time 1 (*β* = 0.10, *p* = 0.015) after controlling for depression at Time 1 (*β* = 0.43, *p* < 0.001) as well. Therefore, *Hypotheses 3.1 and 4.1 were both supported* (see Fig. 1).

According to the approach from Selig and Little (2012)^51^, further analysis by using autoregressive, cross-lagged panel model showed that among Models, Model 5—the reciprocal relationship between PSNU and depression, Model fit: *χ*^*2*^ = 1.185, *df* = 1, CFI = 0.999, NFI = 0.995, RMSEA = 0.018 [0.000, 0.114], SRMR = 0.013—seemed to be the best fitting model in terms of the relationship between PSNU and depression (see the Suppl Appendix).

**Figure 2:**
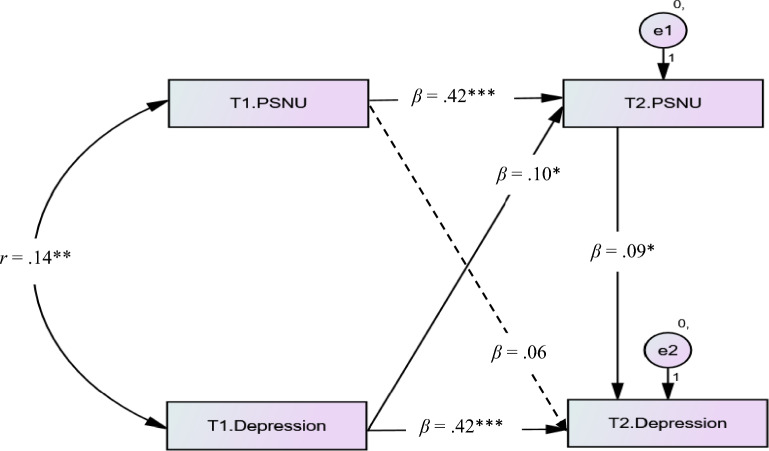
The mediating effect of PSNU at Time 2 in the PSNU at Time 1 and depression at Time 2 link. *PSNU *passive social network usage. The significant paths were showed by the solid lines and the nonsignificant path was showed by the dashed line. T1 = Time 1, T2 = Time 2. *p < 0.050, **p < 0.010, ***p < 0.001.

In addition, PSNU at Time 2 played a fully mediating role in the relationship between PSNU at Time 1 and depression at Time 2 (see Fig. 2). When adding the path from PSNU at Time 2 to depression at Time 2, the effect of PSNU at Time 1 on depression at Time 2 was nonsignificant (*β* = 0.06*, p* = 0.185). Thus, it was concluded that PSNU at Time 1 was linked to depression at Time 2 because of its correlation with PSNU at Time 2.

**Figure 3:**
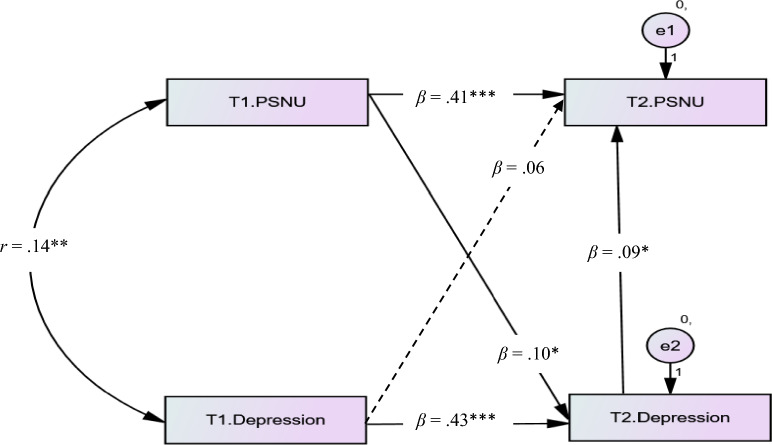
The mediating effect of depression at Time 2 in the depression at Time 1 and PSNU at Time 2 link. *PSNU* passive social network usage. The significant paths were showed by the solid lines and the nonsignificant path was showed by the dashed line. *T1* Time 1, *T2* Time 2. *p < 0.050, **p < 0.010,***p < 0.001.

Similarly, depression at Time 2 also fully mediated the relationship between depression at Time 1 and PSNU at Time 2 (see Fig. 3). After including the path from depression at Time 2 to PSNU at Time 2, the effect of depression at Time 1 on PSNU at Time 2 was nonsignificant (*β* = 0.06, *p* = 0.180). Therefore, it was concluded that depression at Time 1 was linked to PSNU at Time 2 due to its association with depression at Time 2.

### Testing the mediating effect of envy and the cross-lagged model of PSNU and negative emotions

#### Testing the mediating effect of envy

PSNU positively predicted envy (Time 1: *β* = 0.21, *p* < 0.001, Time 2: *β* = 0.22, *p* < 0.001), and envy positively predicted negative emotions (Time 1: *β* = 0.30, *p* < 0.001, Time 2 = *β* = 0.25, *p* < 0.001); the total effect of PSNU on negative emotions without envy was also significant (Time 1: *β* = 0.24, *p* < 0.001; Time 2: *β* = 0.28, *p* < 0.001), and the effect was still significant when envy was added (Time 1: *β* = 0.18, *p* < 0.001; Time 2: *β* = 0.23, *p* < 0.001).

The mediation of envy was positive and significant, indirect effect was estimated, Time 1: = 0.07, 95% CI = [0.04, 0.10] and Time 2: = 0.06, 95% CI = [0.03, 0.09], indicating envy partially mediated the relationship between PSNU and negative emotions at two time points. The effect size of envy was estimated at Time 1 (κ^2^ = 0.061, 95% CI = [0.03, 0.09]) and at Time 2 (κ^2^ = 0.056, 95% CI = [0.03, 0.09]) indicating small to medium effect sizes^49,50^. Therefore, *Hypotheses 2.1 and 2.2 were supported*.

#### Testing the cross-lagged model of PSNU and negative emotions

**Figure 4:**
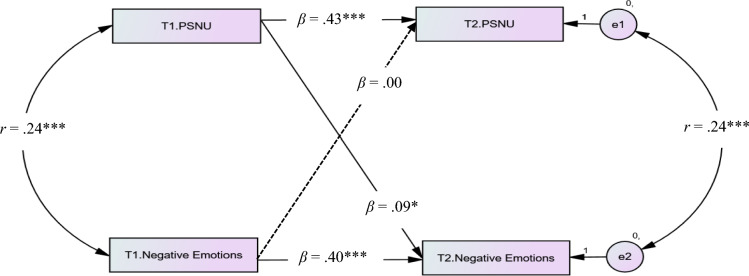
The mediating effect of envy and cross-lagged model of PSNU and negative emotions.* PSNU* passive social network usage, *NE* negative emotions. The significant paths were showed by the solid lines, while the nonsignificant path was showed by the dashed line. *T1* Time 1, *T2* Time 2. *p < .050, ***p < 0.001.

PSNU at Time 2 was predicted by PSNU at Time 1 (*β* = 0.43, *p* < 0.001) but not predicted by negative emotions at Time 1 (*β* = 0.00, *p* = 0.999). Therefore, Hypothesis 4.2* was not supported*. In contrast, negative emotions at Time 2 was predicted by PSNU at Time 1 (*β* = 0.09, *p* = 0.034) after controlling for negative emotions at Time 1 (*β* = 0.40, *p* < 0.001). Therefore, Hypothesis 3.2* was supported* (see Fig. 4).

Similarly, further analysis by using autoregressive, cross-lagged panel model showed that among Models, Model 8—the impact of PSNU on negative emotions, Model fit: *χ*^*2*^ = 0.927, *df* = 2, CFI = 1.000, NFI = 0.997, RMSEA = 0.000 [0.000, 0.066], SRMR = 0.012—fitted the data well compared to the others in terms of the relationship between PSNU and negative emotions (see the Suppl Appendix).

**Figure 5:**
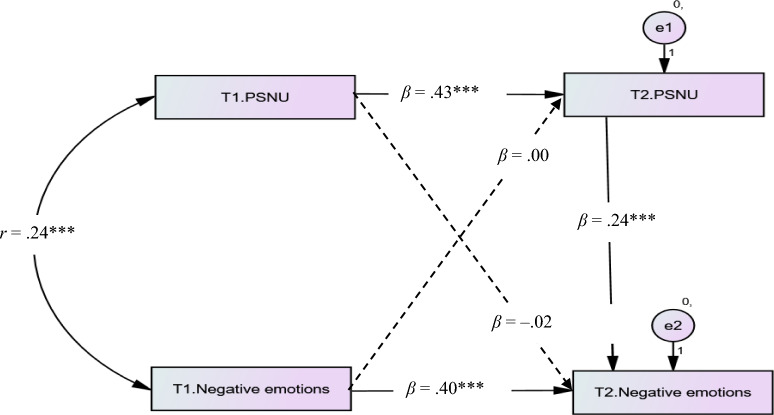
The mediating effect of PSNU at Time 2 in the PSNU at Time 1 and negative emotions at Time 2 link. *PSNU *passive social network usage, *NE* negative emotions. The significant paths were showed by the solid lines, while the nonsignificant paths were showed by the dashed lines. *T1* Time 1, *T2* Time 2. *p < 0.050, ***p < 0.001.

Further analysis indicated that PSNU at Time 2 fully mediated in the relationship between PSNU at Time 1 and negative emotions at Time 2 (see Fig. 5). After PSNU at Time 2 was included, the effect of PSNU at Time 1 on negative emotions at Time 2 was nonsignificant (*β* = –0.02, *p* = 0.652). That is, PSNU at Time 1 was linked to negative emotions at Time 2 because of its association with PSNU at Time 2.

## Discussion

The purpose of the present study is to explore the mediating effect of envy on the relationship between PSNU and depression/negative emotions. By using a longitudinal design, our study offered a deeper understanding of whether such a relationship is reciprocally associated over time.

Regarding the mediating effect of envy in the relationship between PSNU and depression/negative emotions, the results of the present study showed that envy played a mediating role in the relationship between PSNU and depression/negative emotions at the same time (within Time 1 and Time 2), supporting *Hypotheses 1.1, 1.2, 2.1, and 2.2*. According to the social comparison theory^31^, especially upward social comparison, we speculated that when passively using social platforms, users’ envious feelings may be triggered by comparing themselves with friends who are considered having better life than themselves. Therefore, a user’s envy may be linked to increased depression and negative emotions. In addition, engaging in unwanted feelings of envy might further aggravate users’ wellbeing. Other studies revealed that comparing with friends on SNSs might be one of the explanations of the associations between browsing others’ posts and an increase in loneliness feelings^52^ as well as a decrease in positive emotional experience^53^. Hence, our results support previous studies showing that the more time users spent browsing a site passively, the more they increased envious feelings, which, in turn, were linked to depression and negative emotions^10,20,54^.

Regarding the effect of PSNU on depression/negative emotions, consistent with our predictions (*Hypotheses 3.1 and 3.2*), the specific pattern of social network usage, PSNU at Time 1, was related to both depression and negative emotions at Time 2. Furthermore, the aforementioned relationships were fully mediated by PSNU at Time 2. This suggests that PSNU may be a predictor of depression and negative emotions. Our findings align with previous results, indicating that PSNU is associated with wellbeing^21^ and depressed mood over time^54^. Indeed, according to the social comparison theory, Festinger^31^ argued that people prefer objective information to assess their status concerning a certain trait. When such information is unavailable, they would seek social information from others. In the current study, PSNU may serve as social information resulting in negative affections such as negative emotions and depression^37^. Regarding mediator role of envy, previous research has proven its role in the relationship between PSNU and either depression^18^ as well as negative emotion^34^. However, PSNU triggers depression/negative emotions in the simultaneous time point because the link between PSNU at Time 1 and depression/negative emotions at Time 2 were nonsignificant when the mediating role of PSNU at Time 2 was controlled. This means that PSNU at Time 2 plays a vital role in the relationship between PSNU at Time 1 and depression/negative emotions at Time 2 and PSNU can be linked to depression/negative emotions at the same time rather than over time.

Based on the “behavior heuristic”, earlier research showed that when the past behavior is considered as a habit and occurs in stable circumstances, it can result directly in the future behavior^55^. In the current study, PSNU users tended to continue performing the passive pattern of SNSs because this might be their habitual actions (e.g., browsing, no comments, no likes). Thus, their PSNU in the future is more likely to be directly predicted by their PSNU in the past, and the future PSNU serving as stimulating factors is linked to their depression/negative emotions via the cognitive role of envy concurrently.

Regarding the effects of depression/negative emotions on PSNU, it was found that depression at Time 1 was found to be associated with an increase in PSNU at Time 2 (Hypotheses 4.1), and this relationship was fully mediated by depression at Time 2. When individuals recognize inconsistencies between their cognition and behaviors, they strive to eliminate or minimize such dissonance to maintain harmony between their cognition and behaviors^42^, as suggested by the cognitive dissonance theory. The concept of cognitive dissonance also relates to the selective exposure theory^44^. Based on this theory, individuals are prone to favor and focus on information that reinforces or confirms their preexisting perceptions of specific topics while avoiding informative disharmony^21,42^. Therefore, in this paper, when a person experiences depressive moods (cognitive function), they may choose PSNU (behavioral function) as a possible strategy to maintain consistency with their psychological states. In addition, the SNS context immerses depressive users with other people’s interesting lives via browsing their posts and photos passively, which may trigger their depressive moods repeatedly. Additionally, the direct relationship (depression at Time 1 to PSNU at Time 2) disappeared when the effect of depression at Time 2 was included. This could illustrate that past depression with its cognitive functions and negative emotions may have a long-lasting effect on future depression; and the depression in the future time point links to PSNU at the same time point. Indeed, previous study showed that future depression among youth can be predicted by their past depression effectively^56^. In the present study, depression at Time 2, which was associated with depression at Time 1, was linked to PSNU in order to maintain and repeatedly evoke consistent depressive moods. Thus, depression can shape users’ behavior according to the current findings.

Unexpectedly, negative emotions at Time 1 were *not* linked to PSNU at Time 2 (*Hypotheses 4.2*). The emotion-as-a-feedback-system theory also claims that emotional states can affect behavior *indirectly* through cognition, rather than influence behavior *directly*; specifically, this theory emphasizes on anticipated emotion can predict behaviors, and the anticipated emotion is like residues of past emotional reactions and is learnt from the previous experience^57^. In the current study, negative emotions are considered *purely* emotional states by being accessed via upset, scared, or nervous; whereas depression can be characterized by its cognitive elements^23^ and the negative emotions as well^17^. Hence, both negative emotions and negative cognitive thoughts together play significant roles in the depressive individuals’ behavior over time^58^. The current findings confirmed that the higher level of depression, the more frequently the students used social networks passively. However, negative emotions did not have this pattern as did depression.

Indeed, based on the autoregressive models, the results revealed that PSNU and depression was reciprocally associated, wherein PSNU might potentially increase the likelihood of experiencing depression over time, and individuals experiencing depression may also engage in PSNU more frequently. However, the model of impact of PSNU on negative emotions fitted the data best, showing that PSNU might potentially trigger negative emotions but not the other way around, indicating experiencing negative emotions in the short-term might not necessarily lead to PSNU at a later time point.

## Conclusion

In sum, the findings revealed the underlying mechanism where envy may have acted as a mediator in the relationships between PSNU and depression/negative emotions at the two time points. PSNU can predict both inner health issues (depression/negative emotions), but only depression rather than negative emotions can predict specific behavior on SNSs (PSNU). The current study’s findings, which are based on social comparison theory and cognitive dissonance theory, demonstrated the applicability of the two theories in a specific scenario as the reciprocal relationships between PSNU and depression/negative emotions over time. In addition, in the current study, depression, which was considered to include both cognitive and emotional dimensions, had a longer effect on PSNU than negative emotions. Possibly due to the wide range of emotional and physical issues that impact daily function, depression seems to have a more detrimental effect than negative emotions, as it often has longer-term effect on passive networking users.

Some limitations need to be considered. Firstly, because the interval period between the data collections at Time 1 and Time 2 was only two months, “practice effect” may exist so that participants’ responses might depend on memory in some degree. Future studies may consider extending the duration between the two-time measures in order to identify the reciprocal effects. Future studies can adopt more sophisticated design to collect the social network usage data at the third time point to confirm a concrete relationship between PSNU and depression or negative emotions with a more stable effect. Secondly, our sample consisted of high school students in southern Vietnam, so caution should be exercised when drawing conclusions related to the psychological health of other age groups or ethnicities. Future studies could be conducted on different age or ethnic samples to increase the generalizability of findings. Thirdly, active social network usage was proven to have an effect on the depressed mood or anxiety of users^59^. Therefore, in addition to PSNU, future studies regarding SNSs could take into account the effect of both PSNU and active social network usage or their interaction on depression/negative emotions.

Despite the above limitations, the study findings contribute to our understanding of how PSNU may be linked to depression/negative emotions via envy at every time point and how depression may have a long-term effect on the pattern of SNS usage, especially PSNU. Based on these results, we suggest the following: (1) Because envy is a mediator of the relationship between PSNU and depression/negative emotions, this factor should be considered to decrease the negative impact and facilitate PSNU. For example, if users realize that PSNU may likely trigger envious feelings, based on the positive side of upward social comparison^60^, educators or parents may encourage them to make an effort at self-improvement to improve their own performance. (2) Depression may increase PSNU in the future. Depressive individuals may tend to lurk on SNSs without interacting with others in the later time points. This finding raises concerns about the long-term impact of depression on SNS usage and provides practitioners with recommendations for preventing early mental health issues. Overall, the findings can contribute to the clinical and education fields regarding adolescents’ social network site usage and psychological states (i.e., depression and negative emotions). By taking into account the relevant factors of adolescents’ PSNU, intervention and prevention should be designed to both reduce the potential negative effects and boost the positive side of SNS usage. Finally, there is a scarcity of detailed analysis in prior studies^21,54^; thus, the present study provided further analysis after the reciprocal relationships (PSNU and depression) and a directional association from PSNU to negative emotions was found. Therefore, this study offers insights on the bidirectional relationship between users’ behavior on SNSs and depression/negative emotions based on social comparison theory and cognitive dissonance theory.

Our research contributes to a better understanding of the relationship between high school students' social media activities, with the aim of preventing negative health outcomes such as depression and negative emotions. Specifically, we identified the underlying mechanism by which PSNU triggers depression and negative emotions via envy among high school students. These findings have important implications for designing interventions that can help adolescent social media users mitigate the negative effects of PSNU. Overall, our study provides evidence-based suggestions for reducing the harmful impact of PSNU on mental health.

## Supplementary Information

Supplementary Information.

## Data Availability

The authors confirm that the data supporting the findings of this study are available from the corresponding author on reasonable request.
