# Supplementary material for: The association between passive social network usage and depression/negative emotions with envy as a mediator
Source: Sci Rep. 2023 Jun 21;13:10097. doi: 10.1038/s41598-023-37185-y (PMC10284842; doi:10.1038/s41598-023-37185-y)
Supplement: Supplementary file 1 — Supplementary Information. [file 41598_2023_37185_MOESM1_ESM.docx]

**The Association between Passive Social Network Usage and Depression/Negative Emotions with Envy as a Mediator**

Wen Cheng^1^, Duc Nhan Nguyen^2^, and Pham Ngoc Thien Nguyen^3*^

^1^ Center for Teacher Education, Institute of Education Development, & International Graduate Program of Education and Human Development, National Sun Yat-sen University, Kaohsiung, Taiwan (R.O.C.)

^2^ International Graduate Program of Education and Human Development, National Sun Yat-sen University, Kaohsiung, Taiwan (R.O.C.); Ho Chi Minh City University of Education, Ho Chi Minh City, Vietnam

^3^ International Graduate Program of Education and Human Development, National Sun Yat-sen University, Kaohsiung, Taiwan (R.O.C.); An Giang University, Vietnam National University Ho Chi Minh City, Vietnam

^*^ Corresponding author

**Author Note**

We have no conflict of interest to disclose.

Correspondence concerning this article should be addressed to Pham Ngoc Thien Nguyen, International Graduate Program of Education and Human Development, National Sun Yat-sen University, Kaohsiung, Taiwan (R.O.C.). Email: [npnthien@agu.edu.vn](mailto:npnthien@agu.edu.vn)

**Appendix**

**The autoregressive effects of the variables in the cross-lagged model**

**PSNU and Depression**

Table 3 shows that the findings of cross-lagged path analyses. Due the saturation of the initial model, we imposed a constraint on the correlation between PSNU and negative emotions/depression at Time 1, making it equal to the correlation between the errors of PSNU and depression at Time 2, based on their resemblance. This approach provided the models with degrees of freedom, enabling to assess the model fits and the autoregressive effect – the influence of a variable on itself over time. According to the approach from ^49^, the constrained paths without the cross-lagged association between PSNU and depression (Model 2) was not significantly different from the baseline model without cross-lagged association between PSNU and depression (Model 1), indicating the constrain of correlations made no differences, ∆*χ*^2^ _(Model 2 vs Model 1)_ = 1.144, *p* = .285. Second, the PSNU effect model (Model 3) with PSNU at an earlier time point affecting on depression at the later time point fitted the data better than Model 2, ∆*χ*^2^ _(Model 2 vs Model 3)_ = 5.643, *p* = .018; so did the depression effect (Model 4) with depression at an earlier time point affecting on PSNU at the later time point, ∆*χ*^2^ _(Model 2 vs Model 4)_ = 8.228, *p* = .004. Last, the reciprocal effect (Model 5) yielded a better model fit than Model 2 [∆*χ*^2^ _(Model 2 vs Model 5)_ = 13.66, *p* = .001], Model 3 [∆*χ*^2^ _(Model 3 vs Model 5)_ = 8.017, *p* = .005], and Model 4 [∆*χ*^2^ _(Model 4 vs Model 5)_ = 5.432, *p* = .020]. Therefore, the reciprocal effect model (Model 5) was supported by the data, suggesting that reciprocal causal relationship between PSNU and depression existed (see Figure 6).

**Figure 6**

*Autoregressive, Cross-Lagged Path Model (PSNU and Depression)*


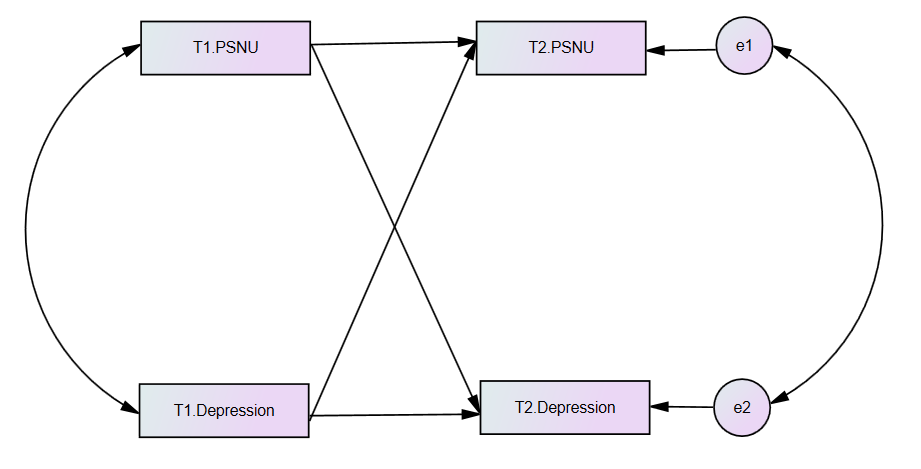


a (+)

b (+)

d (+)

c1 (+)

c2 (+)

e (+)

*Note*. PSNU = passive social network usage. T1 = Time 1. T2 = Time 2. Model 1: Baseline Model without cross-lagged panel (PSNU and depression; d and e paths were both dropped). Model 2: Constrained paths without cross-lagged panel (PSNU and depression; d and e paths were both dropped). Model 3: The PSNU effect (PSNU and depression; e path was dropped). Model 4: The depression effect (PSNU and depression; d path was dropped). Model 5: The reciprocal effect (PSNU and depression; all paths were included).

**Table 3**

*Model Fit Indexes and Comparison of Different Models of the Relationship between PSNU and Depression*

*
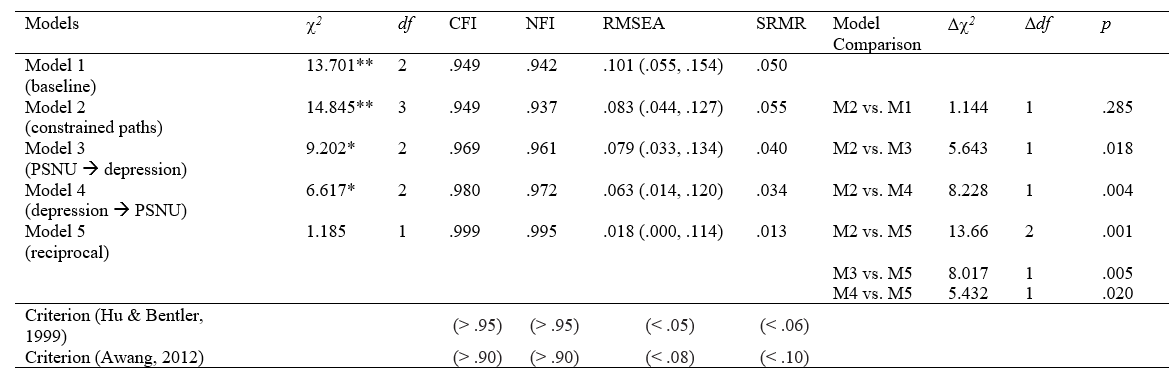
Note*. Model 1 = Baseline Model without cross-lagged panel (PSNU and depression). Model 2 = Constrained paths without cross-lagged panel (PSNU and depression). Model 3 = The PSNU effect (PSNU and depression). Model 4 = The depression effect (PSNU and depression). Model 5 = The reciprocal effect (PSNU and depression).

**PSNU and Negative Emotions**

Table 4 shows that the findings of cross-lagged path analyses. First, the constrained paths without the cross-lagged association between PSNU and negative emotions (Model 7) was not significantly different from the baseline model without cross-lagged association between PSNU and negative emotions (Model 6), indicating that the constrain of correlations made no differences, ∆*χ*^2^ _(Model 7 vs Model 6)_ = 0.707, *p* = .400. Second, the PSNU effect model (Model 8) with PSNU at an earlier time point affecting on negative emotions at the later time point fitted the data better than Model 7, ∆*χ*^2^ _(Model 7 vs Model 8)_ = 4.68, *p* = .031. Third, the negative emotions effect (Model 9) with negative emotions at an earlier time point affecting on PSNU at the later time point did not fit the data better than Model 7, ∆*χ*^2^ _(Model 7 vs Model 9)_ = 0.32, *p* = .572. Last, the reciprocal effect (Model 10) did not yield a better model fit than Model 7 [∆*χ*^2^ _(Model 7 vs Model 10)_ = 4.865, *p* = .088], Model 8 [∆*χ*^2^ _(Model 8 vs Model 10)_ = 0.185, *p* = .667], but was better than Model 9 [∆*χ*^2^ _(Model 9 vs Model 10)_ = 4.545, *p* = .033]. Based on these results, Model 8 (PSNU to negative emotions) seemed to be the best fitting model, suggesting that the path from PSNU to negative emotions, rather than a reciprocal relationship, was present (see Figure 7).

**Figure 7**

*Autoregressive, Cross-Lagged Path Model (PSNU and Negative Emotions)*


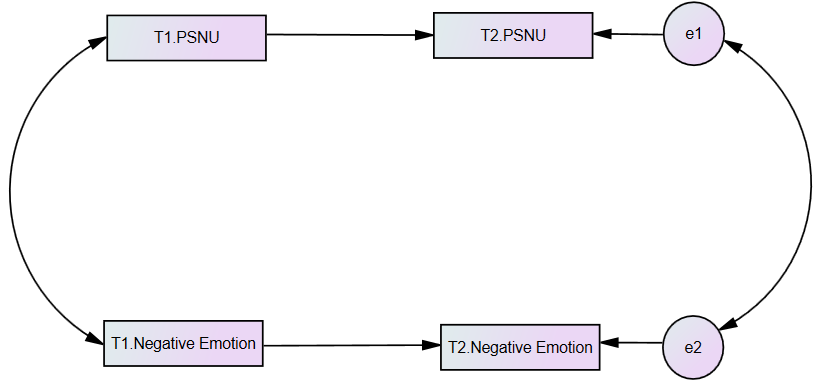


f (+)

g (+)

h1 (+)

h2 (+)

j (+)

i (+)

*Note*. PSNU = passive social network usage. T1 = Time 2. T2 = Time 2. The significant paths were showed by the solid lines. The nonsignificant path was shown by the dashed line. Model 6: Baseline Model without cross-lagged panel (PSNU and negative emotions; i and j paths were both dropped). Model 7: Constrained paths without cross-lagged panel (PSNU and negative emotions; i and j paths were both dropped). Model 8: The PSNU effect (PSNU and negative emotions; j path was dropped). Model 9: The negative emotions effect (PSNU and negative emotions; i path was dropped). Model 10: The reciprocal effect (PSNU and negative emotions; all paths were included).

**Table 4**

*Model Fit Indexes and Comparison of Different Models of the Relationship between PSNU and Negative Emotions*

*
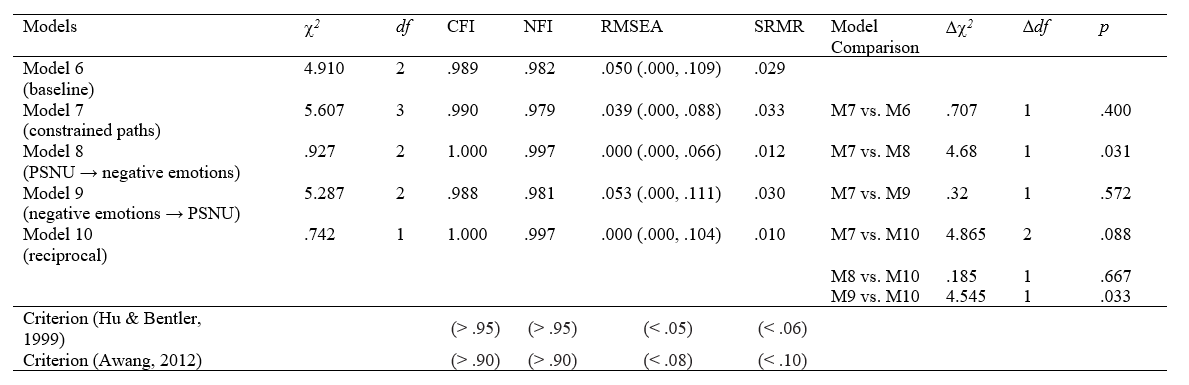
*

*Note*. Model 6 = Baseline Model without cross-lagged panel (PSNU and negative emotions). Model 7 = Constrained paths without cross-lagged panel (PSNU and negative emotions). Model 8 = The PSNU effect (PSNU and negative emotions). Model 9 = The negative emotions effect (PSNU and negative emotions). Model 10 = The reciprocal effect (PSNU and negative emotions).
